# A conceptual framework for nomenclatural stability and validity of medically important fungi: a proposed global consensus guideline for fungal name changes supported by ABP, ASM, CLSI, ECMM, ESCMID-EFISG, EUCAST-AFST, FDLC, IDSA, ISHAM, MMSA, and MSGERC

**DOI:** 10.1128/jcm.00873-23

**Published:** 2023-10-26

**Authors:** Sybren de Hoog, Thomas J. Walsh, Sarah A. Ahmed, Ana Alastruey-Izquierdo, Barbara D. Alexander, Maiken Cavling Arendrup, Esther Babady, Feng-Yan Bai, Joan-Miquel Balada-Llasat, Andrew Borman, Anuradha Chowdhary, Andrew Clark, Robert C. Colgrove, Oliver A. Cornely, Tanis C. Dingle, Philippe J. Dufresne, Jeff Fuller, Jean-Pierre Gangneux, Connie Gibas, Heather Glasgow, Yvonne Gräser, Jacques Guillot, Andreas H. Groll, Gerhard Haase, Kimberly Hanson, Amanda Harrington, David L. Hawksworth, Randall T. Hayden, Martin Hoenigl, Vit Hubka, Kristie Johnson, Julianne V. Kus, Ruoyu Li, Jacques F. Meis, Michaela Lackner, Fanny Lanternier, Sixto M. Leal Jr., Francesca Lee, Shawn R. Lockhart, Paul Luethy, Isabella Martin, Kyung J. Kwon-Chung, Wieland Meyer, M. Hong Nguyen, Luis Ostrosky-Zeichner, Elizabeth Palavecino, Preeti Pancholi, Peter G. Pappas, Gary W. Procop, Scott A. Redhead, Daniel D. Rhoads, Stefan Riedel, Bryan Stevens, Kaede Ota Sullivan, Paschalis Vergidis, Emmanuel Roilides, Amir Seyedmousavi, Lili Tao, Vania A. Vicente, Roxana G. Vitale, Qi-Ming Wang, Nancy L. Wengenack, Lars Westblade, Nathan Wiederhold, Lewis White, Christina M. Wojewoda, Sean X. Zhang

**Affiliations:** 1 Radboudumc-CWZ Centre of Expertise for Mycology, Nijmegen, the Netherlands; 2 Foundation Atlas of Clinical Fungi, Hilversum, the Netherlands; 3 Department of Dermatology and Venereology, Peking University First Hospital, Beijing, China; 4 Department of Basic Pathology, Federal University of Paraná, Curitiba, Brazil; 5 Research Center for Medical Mycology, Peking University, Beijing, China; 6 International Society for Human and Animal Mycology (ISHAM), Working Group Nomenclature; 7 Center for Innovative Therapeutics and Diagnostics, Richmond, Virginia, USA; 8 University of Maryland School of Medicine, Baltimore, Maryland, USA; 9 Nomenclature Committee for Fungi, International Mycological Association (IMA); 10 Fungal Diagnostics Laboratory Consortium (FDLC); 11 Mycoses Study Group, Education and Research Consortium (MSG-ERC); 12 European Confederation of Medical Mycology (ECMM); 13 Clinical and Laboratory Standards Institute (CLSI); 14 Medical Mycological Society of the Americas (MMSA); 15 ISHAM Working Group on Diagnostics; 16 Mycology Reference Laboratory, Spanish National Centre for Microbiology, Madrid, Spain; 17 Fungal Infection Study Group, European Society of Clinical Microbiology and Infectious Diseases (EFISG/ESCMID), Basel, Switzerland; 18 Departments of Medicine and Pathology, Duke University, Durham, North Carolina, USA; 19 Department of Microbiology and Infection Control, Statens Serum Institut, Copenhagen, Denmark; Department of Clinical Microbiology, Copenhagen University Hospital Rigshospitalet, Copenhagen, Denmark; 20 Antifungal Susceptibility Testing Subcommittee of European Committee of Antimicrobial Susceptibility Testing (EUCAST-AFST); 21 Clinical Microbiology Service, Department of Pathology and Laboratory Medicine, Memorial Sloan Kettering Cancer Center, New York, USA; 22 Mycology Committee of Chinese Society for Microbiology; 23 Institute of Microbiology, State Key Laboratory of Mycology, Chinese Academy of Sciences, Beijing, China; 24 Medical Mycology Society of Chinese Medicine and Education Association; 25 Asia Pacific Society for Medical Mycology; 26 ISHAM Working Group Veterinary Mycology and One Health; 27 Mycological Society of China (MSC); 28 Clinical Microbiology at The Ohio State University Wexner Medical Center, Columbus, Ohio, USA; 29 National Mycology Reference Laboratory, Public Health England, Bristol, United Kingdom; 30 Department of Microbiology, National Reference Laboratory for Antimicrobial Resistance in Fungal Pathogens, Medical Mycology Unit, Vallabhbhai Patel Chest Institute, University of Delhi, Delhi, India; 31 University of Texas Southwestern Medical Center, Dallas, Texas, USA; 32 Division of Infectious Diseases, Mount Auburn Hospital, Cambridge, Massachusetts, USA; 33 Infectious Diseases Society of America (ISDA); 34 University of Cologne, Faculty of Medicine, Institute of Translational Research, Cologne Excellence Cluster on Cellular Stress Responses in Aging-Associated Diseases, Cologne, Germany; 35 Department I of Internal Medicine, University of Cologne, Excellence Center for Medical Mycology, Cologne, Germany; 36 Alberta Precision Laboratories, Public Health Laboratory, Calgary, Alberta, Canada; 37 Mycology Department, Laboratoire de Santé Publique du Québec, Institut National de Santé Publique du Québec (INSPQ), Sainte-Anne-de-Bellevue, Québec, Canada; 38 Department of Pathology and Laboratory Medicine, London Health Sciences Center, London, Ontario, Canada; 39 Department of Mycology, Centre Hospitalier Universitaire de Rennes, Rennes, France; 40 University of Texas Health Science Center, San Antonio, Texas, USA; 41 Clinical and Molecular Microbiology, Department of Pathology, St. Jude Children’s Research Hospital, Memphis, Tennessee, USA; 42 Department of Parasitology (Charité), Institute of Microbiology and Hygiene, Humboldt University, Berlin, Germany; 43 Onoris, École Nationale Vétérinaire, Agroalimentaire et de l'Alimentation Nantes-Atlantique, Nantes, France; 44 Infectious Disease Research Program, Department of Pediatric Hematology and Oncology and Center for Bone Marrow Transplantation, University Children’s Hospital, Münster, Germany; 45 Laboratory Diagnostic Center, RWTH Aachen University Hospital, Aachen, Germany; 46 University of Utah School of Medicine, Salt Lake City, Utah, USA; 47 Loyola University Health System, Loyola University Chicago, Maywood, Illinois, USA; 48 Royal Botanic Gardens, Kew, Richmond, Surrey, United Kingdom; 49 Natural History Museum, London, United Kingdom; 50 University of Southampton, Southampton, United Kingdom; 51 Jilin Agricultural University, Chanchung, China; 52 General Committee for Nomenclature, International Botanical Congress (IBC); 53 Advisory Board of International Commission on the Taxonomy of Fungi (ICTF); 54 Division of Infectious Diseases, Medical University of Graz, Graz, Austria; 55 Translational Medical Mycology Research Unit, ECMM Excellence Center for Medical Mycology, Medical University of Graz, Graz, Austria; 56 European Hematology Association, Specialized Working Group for Infections in Hematology, The Hague, the Netherlands; 57 Department of Botany, Charles University, Prague, Czechia; 58 Clinical Microbiology Laboratory, UMMC Laboratories of Pathology, University of Maryland School of Medicine, Baltimore, Maryland, USA; 59 Public Health Ontario, Toronto, Ontario, Canada; 60 Department of Laboratory Medicine and Pathobiology, Canada and University of Toronto, Toronto, Ontario, Canada; 61 Institute of Hygiene and Medical Microbiology, Medical University of Innsbruck, Innsbruck, Austria; 62 Institut Pasteur, Paris, France; 63 University of Alabama at Birmingham, Birmingham, Alabama, USA; 64 Centers for Disease Control and Prevention, Atlanta, Georgia, USA; 65 Dartmouth Health, Lebanon, New Hampshire, USA; 66 National Institute of Allergy and Infectious Diseases, National Institutes of Health, Bethesda, Maryland, USA; 67 Westerdijk Fungal Biodiversity Institute, Utrecht, The Netherlands; 68 University of Pittsburgh Medical Center, Pittsburgh, Pennsylvania, USA; 69 University of Texas Health Science Center at Houston, Houston, Texas, USA; 70 Clinical Microbiology Laboratory, Wake Forest Baptist Medical Center, Winston-Salem, North Carolina, USA; 71 The American Board of Pathology, Tampa, Florida, USA; 72 American Board of Pathology (ABP); 73 National Mycological Herbarium, Ottawa Research and Development Centre, Science and Technology Branch, Agriculture & Agri-Food Canada, Ottawa, Ontario, Canada; 74 Cleveland Clinic Lerner College of Medicine, Case Western Reserve University, Cleveland, Ohio, USA; 75 Department of Laboratory Medicine, Cleveland Clinic, Cleveland, Ohio, USA; 76 Infection Biology Program, Lerner Research Institute, Cleveland Clinic, Cleveland, Ohio, USA; 77 Beth Israel Deaconess Medical Center, Boston, Massachusetts, USA; 78 Lewis Katz School of Medicine, Temple University, Philadelphia, Pennsylvania, USA; 79 Mayo Clinic, Rochester, Minnesota, USA; 80 Hippokration Hospital, Thessaloniki, Greece; 81 Microbiology Service, Department of Laboratory Medicine, Clinical Center, National Institutes of Health, Bethesda, Maryland, USA; 82 Vanderbilt University Medical Center, Nashville, Tennessee, USA; 83 Consejo Nacional de Investigaciones Científicas y Tecnológicas (CONICET), Buenos Aires, Argentina; 84 Unidad de Parasitología, Sector Micología, Hospital J.M. Ramos Mejía, Buenos Aires, Argentina; 85 Engineering Laboratory of Microbial Breeding and Preservation of Hebei Province, School of Life Sciences, Institute of Life Sciences and Green Development, Hebei University, Baoding, China; 86 Department of Pathology and Laboratory Medicine, Weill Cornell Medical College, New York, USA; 87 Public Health Wales Microbiology, Cardiff, United Kingdom; 88 Department of Pathology and Laboratory Medicine, University of Vermont Medical Center, Burlington, Vermont, USA; 89 Johns Hopkins University School of Medicine, Baltimore, Maryland, USA; Vanderbilt University Medical Center, Nashville, Tennessee, USA

**Keywords:** nomenclature, taxonomy, fungi

## Abstract

The rapid pace of name changes of medically important fungi is creating challenges for clinical laboratories and clinicians involved in patient care. We describe two sources of name change which have different drivers, at the species versus the genus level. Some suggestions are made here to reduce the number of name changes. We urge taxonomists to provide diagnostic markers of taxonomic novelties. Given the instability of phylogenetic trees due to variable taxon sampling, we advocate to maintain genera at the largest possible size. Reporting of identified species in complexes or series should where possible comprise both the name of the overarching species and that of the molecular sibling, often cryptic species. Because the use of different names for the same species will be unavoidable for many years to come, an open access online database of the names of all medically important fungi, with proper nomenclatural designation and synonymy, is essential. We further recommend that while taxonomic discovery continues, the adaptation of new name changes by clinical laboratories and clinicians be reviewed routinely by a standing committee for validation and stability over time, with reference to an open access database, wherein reasons for changes are listed in a transparent way.

## COMMENTARY

### Development of the modern naming system in medical mycology

Advanced and novel diagnostic and research methods, particularly nucleic-acids sequencing, have revolutionized microbial taxonomy. Since rearrangements in the Tree of Life are closely linked to the names of the newly recognized entities, name changes are inevitable. This happens everywhere in microbial taxonomy and is the result of scientific progress. In the kingdom *Fungi*, taxa that were previously delineated by morphological features or physiological profiles are being recharacterized by sequence data underlying their phylogenetic positions. However, unlike most prokaryotic microbes, fungi exhibit a wealth of distinguishing phenotypic characteristics which have served as the basis for accruing clinical data over the centuries. Many fungi have a complicated life cycle, with one or more asexual and sexual forms of sporulation that have very different appearances and are produced under different growth conditions. In the past, it was often not known that these different forms, or morphs, belonged to the same species. This problem was mitigated by the development of a naming system with two separate categories: one name for the sexual morph (teleomorph), and one (or more) for each asexual morph (anamorph). As sexuality is mostly expressed in the environment, and asexual morphs are often preponderant *in vitro* and in the animal host, the connection between the two or more life forms remained problematic despite increasing availability of DNA data, awaiting experimental establishment. Taxonomic categories above the species level were based on the sexual morph and prioritized, while the separate system for asexual ones was generally acknowledged to be artificial—although some authors also published names of families and even higher ranks based only on asexual morphs. Although imperfect, abandoning the knowledge that has been accrued during 270 years of mycological research under this “old” nomenclature system is more painful than in other areas of research, particularly as the old nomenclature is critically linked to patient management.

### Challenges and limitations of molecular taxonomy for medically important fungi

With DNA sequencing and other non-microscopic methods, able to firmly establish species, the system with dual names became increasingly obsolete. After some 20 years of debate, and strongly divided opinions voiced by a Special Committee on alternate names (1), followed by a symposium “One Fungus One Name” held in Amsterdam, in 2011, many of the mycological community came to a consensus to abandon the classical separate naming of different morphs of the same species with the “Amsterdam Declaration” (2). Subsequently, this new direction was formally proposed on the debate “floor” by Redhead (3), disputed, and, finally, adopted by the international community at the International Botanical Congress held in Melbourne in 2011. The change was effective from 2011 and was retro-active (4). This enabled a closer representation of the natural system than phenotypic characterization. However, since many names used in medical and veterinary mycology were in the asexual-based artificial system, the need arose for a fundamentally different approach in systematization. Formal procedures were introduced to keep the number of changes within limits through protected lists of names developed by working groups operating under the auspices of the International Commission on the Taxonomy of Fungi. The ensuing name changes of the combined transition toward molecular phylogeny and priority of the oldest name irrespective of sexual states are dramatic, leaving few names unaffected among the medical fungi. Comparing the second and fourth editions of the *Atlas of Clinical Fungi*, of the names accepted in 2020 (5), only 30% were still used in the same sense as in 2000. This demonstrates an overwhelming impact of molecular taxonomy on medical mycology, larger than any other discipline within clinical microbiology practice.

Numerous authors have expressed their concerns about the pace of name change in mycology and provided lists of recommended names primarily focused on the species level (6 – 10), while others recommended to accept and adopt generic name changes (11 – 13). The present paper aims to mitigate some of these problematic shifts with proposals, which we think are practicable and minimally disruptive for the advancement of science. Certainly, naming of medically important fungi needs a thorough renovation. In many historical handbooks and guidelines, grand subdivisions are based on obsolete morphological criteria, such as “*Dematiaceae*,” a family designation for fungi with often large conidia and conidiophores carrying melanin in the cell wall, or “*Coelomycetes*,” a class rank name for those forming asexual cup- or flask-like spore-bearing structures anywhere in their asexual life cycle.

Molecular phylogeny has enabled major advances in providing logical coherence between taxonomic entities (14) and has led to better understanding of the origin, relationships, and properties of fungi—including whether they are likely to be pathogens of concern or mycotoxin producers. Phylogeny sheds light upon such potentially shared ecological strategies between species. Whenever obvious benefits of the new naming system exist, name changes are easily accepted by stakeholders in the medical community. An example of one such easy adoption is the change of the intracellular pathogen *Talaromyces marneffei* separated from the strictly saprobic *Penicillium* species (15). The natural classification of dermatophytes (16) also met limited resistance. Another example is the segregation of morphologically similar, but phylogenetically extremely remotely related (i.e., ascomycetous versus basidiomycetous) fungi, now placed in separate genera [*Geotrichum/Trichosporon* (17); *Sporothrix/Quambalaria* (18)]. A more recent example can be found in the replacement of the genus name *Phialemoniopsis/Phialemonium* by *Thyridium* which turned out to be the name given earlier to the sexual morph (19).

However, reestablishing the correct systematic position of tens of thousands fungal names cannot be done by a simple declaration, and in some groups of fungi, molecular data is not easy to obtain. The above-mentioned, well-accepted examples share their application of a holistic, biological approach to taxonomy, demonstrating that the newly separated groups are fundamentally different in life cycle, habitat choice and clinically relevant parameters, with molecular phylogeny as a supporting feature facilitating definite identifications. This demonstrates an optimal approach to reach meaningful taxonomic name changes in mycology. In contrast, resistance against change invariably entails cases where the change involves a relatively homogeneous group without clear character difference, which is divided exclusively based on phylogeny. An example is the rearrangement of the ecologically similar (20) genera *Curvularia* and *Bipolaris* where the multi-character phenotypic separation did not match with molecular barcoding (21). Conversely, species of *Chaetomium* were assigned to several novel genera (22). The latter study made these rearrangements with a data set containing strains from indoor habitats alone, implying that criteria other than phylogeny were insignificant; such cases should be reconsidered using a wider range of named species and ecologies.

We advocate that name changes of medically important fungi should be meaningful and carefully applied to reduce the potential for confusion by those responsible for patient care. Name changes are effective and will reach wide application only when based on fundamental differences that have shaped evolution and have clinical relevance. Two levels of diversity, i.e., that of the species and of the genus, have entirely different drivers (7, 8) which are discussed separately, i.e., the subdivision of a species, and the rearrangement and splitting of genera.

### Species diversity: The fragmenting epithet

The borderline between species is not clear-cut, and there are over 30 different species concepts used across biology (23). The classical biological concept is not directly applicable to microbes. Genetic composition and viability of progeny may vary anywhere between 0% and 100% due to all sorts of constraints. Sexually hyperactive strains are able to mate beyond established species borders (24, 25), and this is recognized as hybridization in some plant pathogenic fungi where hybrid nomenclature has been introduced. Conversely, progeny may lack genetic recombination (26 – 28) or may produce sterile hybrids unable to mate (29, 30). Crosses appearing to be sexual may be uniparental, underlining that fungi are able to propagate asexually over extended periods. Many fungi have alternative sexual strategies, which do not require an opposite mating partner (31). Ideally, a species is genetically focused by genetic interaction, but most fungi multiply parts of their life cycle clonally to increase successful genotypes. Asexual reproduction may be dominant, and sexuality often remains cryptic.

The near-absence of sexuality leads to fragmentation of the species into numerous molecular siblings. In taxonomic practice in fungi, the siblings are recognized by mutations in sections of barcoding loci, such as the rDNA ITS regions, *CAM, rPB1, rPB2, TEF1,* or *TUB2* (*BenA*) (32). Genealogical concordance enables *in silico* detection of sexual recombination, providing an operational criterion to verify the species borderline. In taxonomic practice of large data sets, however, most authors use concatenated barcoding sequences to provide molecular distances that are judged sufficient for novel species description. For example, depending on the genes studied, numerous siblings were observed within the genera *Cladosporium* (33) distinguishing 54 siblings in the *C. cladosporioides* complex and *Fusarium* (34) distinguishing 74 siblings in the *F. fujikuroi* complex. In plant pathogens, clonal expansion may occur after horizontal gene transfer of pathogenicity islands (35) leading to lineages, which are sometimes known as special forms. These emerging genotypes may be sampled more frequently as a result and have a higher chance of being detected, giving the false impression of obligate host adaptation. However, the responsible horizontally transferred accessory chromosomes can be dispensable (36), and the species can infect another susceptible host, as in the case of the banana-fruit infecting pathogen *Fusarium musae* that has been encountered in human infection and suggested as a possible health concern (37 – 39).

Decreased sexuality leads to higher clonal diversity (40, 41). An example from clinical fungi is given by dermatophytes, which lose sexuality upon adaptation to the human host, and whose mating types evolve differentially (42, 43). Conversely, numerous genotypes without ecological or clinical differentiation may emerge within a single species (44). Thus, phylogenetic distance in a particular gene region alone is not always a sufficient criterion for novel species description. Distinction of clones based only on molecular difference answers epidemiological questions and is essential to reveal sources of contamination and routes of infection. Speciation, however, is a slower process, with slight differences in ecological preferences of drivers of future separation that ultimately leads to loss of recombination ability. Many barcoding markers are not transcribed and do not result in different evolutionarily relevant properties; they just are a proxy of relevant differences elsewhere in the genome. Addition of more genes should increase the stability of the tree (45).

Species are an amalgamation of genetically deviating lineages, bordered by increasing inability to recombine and produce viable progeny. In contrast to the phenotypic approach, molecular data enable recognition of each lineage. Rather than species, the concept of “species complex” has become a trend. A complex combines molecular siblings without known gene flow under a single umbrella. In most fungal groups, the potential ability of hybridization and recombination between siblings has not been tested. Multi-locus analysis of non-transcribed markers provides potential diversity which is close to infinite (46). Novel names for the individual lineages may conceal the close affinity to the classical species of the aggregate.

### Suggestions for the stability of species names in medical mycology

Given the above-described fragmentation of species into named molecular siblings and to promote stability of species names, taxonomy should be separated from epidemiology. Taxonomy and epidemiology both involve microbial diversity and overlap because species are distinguished from intraspecific lineages genetic separation which is difficult to establish. Rules of botanical nomenclature such as on typification need to be evaluated retrospectively and numerous debatable cases will appear (47). In addition, medical mycology covers only a very small fragment of global biodiversity studies, and endeavoring to impose rules from a medical perspective to the much larger study areas in agriculture, ecology, and industry and involving the entire fungal kingdom is unproductive. Rather, we would suggest adoption of the following recommendations:

- For closely related entities, official nomenclatural categories such as subspecies, variety, and form are available. Individual clones and genotypes below the concordant species level are preferably numbered rather than named (48) as long as they do not fulfill a number of criteria that are in use to define species (outlined above). This enhances the link to the overarching species name and facilitates the connection with existing literature.
- For the description of closely related species, data besides phylogenetic distance, such as the absence of recombination by genealogical concordance, and presence of phenotypic, ecological, clinical, or evolutionarily relevant parameters should be included.
- The species complex, also known as a species aggregate or series, is a practical recommendation rather than a solid scientific conclusion and, thus, may be subject to improved circumscription, covering more siblings defined with the ex-type strain as reference.
- To establish the classical species as the portal toward all existing literature, it is recommended that the overarching species complex name is always mentioned, followed by the sibling’s or cryptic species name.
- As species names are linked to (epi)type material as the ultimate reference, this should be accessible to investigation, and hence, deposition of a living culture in a reference collection should become mandatory in mycology for those fungi that can be cultured; if the type is a drawing or in inaccessible fungarium or deteriorated herbarium material and, therefore, unrecognizable molecularly, epi-typification or deposition of a reference culture is strongly encouraged.
- Mycologists introducing changes near the species level should be required to provide criteria with which the species at hand can be recognized, i.e., a diagnosis, as is already recommended in the *Code*.
- Name changes generally acquire wide recognition only after the underlying taxonomy has been confirmed in peer-reviewed papers by separate groups of researchers using trees with different taxon sampling. Name stability and validation should be held to the highest possible standard for medically important fungi where clinical decisions based upon that information directly influence patient management.
- As taxonomic science progresses, a committee composed of clinical microbiologists, physicians, medical mycologists, and taxonomists would review proposed name changes for medical relevance, validity, and stability for adoption in clinical laboratories and patient care.

### Genus diversity: the phylogenetic framework

At the genus level, the problems are entirely different. While species are subdivided because diverse entities are distinguishable by modern methodology, at the genus level, the central question is the position in the phylogenetic Tree of Life. To this aim, markers with a lower mutation rate are used, particularly those of the ribosomal repeat. Numerous methods have been developed to optimally reflect the course of evolution (49). Different gene trees are expected to be concordant albeit with different resolution. Reclassification of a fungus leads to a new combination of a genus name where the original species epithet is maintained. Consequently, generic rearrangements have become a major source of naming instability.

In contrast to the species, there are no operational molecular criteria to define and circumscribe a genus. All taxonomic entities above the species level can be introduced at will. Indeed, different traditions in the respective research areas have led to genera enormously deviating in size and level of intrageneric diversity. Construction of phylogenetic trees has become the nearly exclusive approach in establishing the position and size of genera. However, as phylogenetic trees are fundamentally relative, being based on mutual comparison of its members, generally using limited numbers of barcoding gene regions which are for diagnostics rather than for taxonomy, they suffer from inherent instability during the early years of molecular exploration. Although methods of tree reconstruction are highly sophisticated, the underlying taxon sampling effect causes variation with every selection of objects in the tree. Sampling may be relatively complete in well-known groups with a long history of research, but a balanced overview of extant diversity concerns selected groups with practical significance rather than complete genera.

In common taxonomic practice, the main generic parameter is phylogenetic distance. In this approach, genera are statistically supported aggregates of similar species as recognizable clades by conserved, single or concatenated markers. Without analysis of concordance of genes, molecular phylogeny alone is one-dimensional, similar to the obsolete approach of using only microscopic morphology. Different levels of diversity have largely been determined by the taxonomic history of the genus at hand. Many newly created genera are clades that contain just a few species. Rearrangement of such clades requires creation of other genera; small genera comprising just a few species, thus, have a large risk of further fragmentation. As an example, *Scopulariopsis* and *Microascus* were previously distinguished as being asexual or sexual, respectively; presently, the names are used for two groups that are molecularly distinct, with a small genus *Pithoascus* between the clades that contain the type species of the two genera (50). Phenotypic descriptions of *Scopulariopsis* and *Microascus* in the new concepts are nearly identical. In such a case, synonymy of *Pithoascus* with the oldest generic name, *Microascus*, would have required just a few name changes of rare fungi and may be a more pragmatic approach. Another example is the afore-mentioned separation of *Curvularia* and *Bipolaris*, where phylogeny did not match with classical phenotypes. Prioritizing phylogeny, Manamgoda et al. (21) reshuffled species of both genera, but a more parsimonious solution would have been the recognition that the bipartition apparently does not exist, giving priority to the single genus name *Curvularia*.

In contrast, the large genus *Aspergillus* with its characteristic conidiophores was recognized as a group since its establishment in 1809 and now comprises numerous smaller clades. The genus is monophyletic (51), but distances between ultimate members of the genus clade are much larger than usual. In addition, fungi without the characteristic aspergillus-like conidiophores may appear to contain similar genotypes, leading to merging of such genera (e.g., *Phialosimplex, Polypaecilum*) with *Aspergillus* (52). Broader generic circumscriptions cover smaller ones; genera then do not fall apart but tend to become larger. In general, it may be nomenclaturally advantageous to maintain broad generic concepts for taxa like *Aspergillus, Chaetomium*, and *Fusarium* in their classical sense, as long as they are monophyletic. Smaller genera will appear synonymous, but this usually involves a lower number of name changes.

In the yeasts, classical genera are mostly not monophyletic. The clinically relevant genera that have been classified in *Candida* on the basis of physiological criteria, phylogenetically were found to belong to eight families (5), and therefore, the genus in the traditional sense is untenable. Some genera could coincide with the current level of family ranks, e.g., *Debaryomycetaceae* containing the single genus *Candida* which might keep future instability within limits.

Genera will be meaningfully distinguished when the clades are consistently supported by high bootstrap values. This was successful with the dermatophytes mentioned above (16), but statistical support of the backbone of trees is often lacking or minimal. For example, the family *Herpotrichiellaceae* (black yeasts and allies) in *Chaetothyriales* has been analyzed repeatedly using different genes and data sets but achieving poor statistical support below the family level (53). The authors decided to leave the genera defined by morphology despite the known disagreement with phylogeny. This provides temporary nomenclatural stability, but such genera remain highly polyphyletic, and treating unrelated species under the same generic name, as done, e.g., by Thitla et al. (38) for *Exophiala*, cannot be recommended in the long run.

There may be an optimal size of genera. Extensive divisions resulting in unique genera for many medically important species risks loss of phylogenetic coherence and ease of recognition. On the other hand, it is difficult to develop diagnostic criteria for genera that are too broad.

### Suggestions for stability of the genus concept in medical mycology

- Genera are preferably based on phenotypic characteristics supplemental to molecular distance, reflecting main ecological and medical significance, or evolutionary or other behavioral trends, such that their separation is likely to be widely understood and accepted.
- Names of large-sized genera such as *Aspergillus* tend to be more stable than small genera containing just a few species, based upon phylogenetic distance alone; provided that the genus is monophyletic, it is recommended to maintain the size of genera if there is no compelling reason to break them up.
- Small and phenotypically similar genera in the same clade can be combined if this reduces the number of necessary name changes.
- Authors introducing name changes at the generic level are requested to provide or select criteria with which the genus at hand can be recognized.
- Name changes are preferably adopted in routine practice only after the underlying taxonomy has been confirmed in several papers by independent authors; an evaluating Committee under the auspices of ISHAM is preferable.
- Remember that there are procedures under the *Code* to avoid the necessity to replace a well-known genus name by a less known one, using a conservation process, while also a change of the original type species is possible.

## DISCUSSION

Taxonomy is not a galactic spaceship operating in a scientific vacuum. On the contrary: thousands of diagnostic laboratories worldwide in all areas of microbiology apply the outcome of this research on a daily basis for patient care. There are few areas of fundamental science with comparable practical implications. Nomenclatural changes should, therefore, be scientifically sound and based on convincing evidence (54, 55), taking into account phenotypic, biological, and ecological features as well as clinically relevant characteristics of pathogenicity and specifically antifungal resistance patterns. In the absence of established scientific criteria for delimiting genera, the default should be that proposed reclassification should benefit the clinical user. An ongoing discussion relevant to medical mycology concerns the bipartition of *Fusarium* in *Fusarium s.str*. and *Neocosmospora*. The latter is an interesting showcase of the consequences of generic fragmentation. Although the phylogenetic distance between *Neocosmospora* and *Fusarium* may justify their separation, it necessitates the maintenance of 21 additional fusarium-like genera, which are phylogenetically more distant from *Fusarium s. str*. than *Neocosmospora*. The separation of *Neocosmospora* is not necessary for the medical community to be adopted immediately; this can wait until taxonomists stabilize the classification over years of investigation and consensus. A similar appeal was made by Redhead (56), who in proposing a reclassification of coprinus-like species, stated that we do not need to adopt these names immediately; instead, we may be conservative initially. Following molecular testing by different laboratories, the pathogens named from asexual cultures, *Hormographiella aspergillata* and *H. verticillata*, were subsequently synonymized with the mushroom species *Coprinopsis cinereus* and *Coprinellus domesticus*, respectively.

At the species level, we are able to show massive amounts of difference below the level of genetic exchange, which remains the ultimate criterion for conspecificity (organisms belonging to the same species). Although this borderline is vague, subject to many exceptions, and often difficult to establish in research areas outside microbiology, it is uncommon to attribute species status to interbreeding entities. In several groups, more entities can be distinguished than is taxonomically meaningful. In dermatophytes, Tang et al. (48) made a recommendation to formally name only those siblings that are relevant to clinical practice. For example, Bian et al. (57) synonymized several of the described entities in the *Aspergillus niger* clade and Sklenář et al. (58) in the *A. versicolores* clade. The requirement to distinguish large numbers of molecular siblings without clinical relevance would have a negative impact on clinical practice and patient care when the siblings are only recognizable by experimental, non-microscopic methods such as multi-locus sequencing and have no broader significance.

How should a species name be reported? For routine clinical practice, it has been claimed that identification at species complex level is usually sufficient (6, 9) although this may depend on the fungal group at hand or the clinical questions (59). Sequencing or MALDI-ToF MS (Matrix Assisted Laser Desorption/Ionization coupled to time-of-flight mass spectrometry) may directly identify the sibling name, without being aware that this is a member of a particular species complex. As MALDI-ToF MS results may be dependent on the spectral database used for comparison, it is recommended to mention this database with version number along with the identification in the lab report for retrospective analysis. Since nearly all major pathogenic species comprise subspecific entities, both the species complex and the sibling should be identified in clinical laboratory reports, with the siblings reported either by name or lineage number as applicable. Reporting would, therefore, appear as follows: *Trichophyton interdigitale* (member of *T. mentagrophytes* species complex), or, in case of an unnamed lineage: *Trichophyton mentagrophytes* species complex (molecular sibling ITS XIV). For the numerous species that are not part of a complex, the single recommended name is sufficient. Given the fact that the size of a genus lacks clear parameters, in practice for some fungi, several names may remain in use concomitantly until phylogenetic disputes can be resolved, as highlighted by the example of *Fusarium/ Neocosmospora* (60 – 63).

At times, this situation may become very confusing. For example, *Mucor elegans* is synonymous with *Actinomucor elegans* and *Rhizopus elegans*, but *Apophysomyces elegans* is a completely different species. One solution to reduce naming confusion is to provide an easily usable and complete tool where current and prior names can be found and mapped to which species complex they belong. A list of currently recommended names for medical fungi is available open access at www.atlasclinicalfungi.org/, subheader nomenclature, also providing synonyms and affiliation to species complexes. The database has a comfortable search function as well as a printable version of the list recommended names. Use of the database prevents the necessity to mention old and new names in clinical reports. A committee of clinical microbiologists, physicians, and medical mycologists has been formed under the auspices of the International Society for Human and Animal Mycology (ISHAM), Working Groups on Nomenclature and Fungal Diagnostics, where names will be discussed, reviewed, and adjudicated for medical relevance, validity, and stability, consulting specialists on the topic. Recommendations will be publicized for transparency. We strongly recommend deposition of representative strains in the established fungal culture collections for future investigation.

The stability of names remains a much-debated issue, and there is no simple solution. Names will inevitably change. However, the changes should have some benefit. There are ways to mitigate some of the detrimental effects, but recommendations tend to have only temporary effect. As a recommendation to taxonomists, we suggest that rules in the *Code* (64, 65) could be used more than they are now to promote stability. For example, changing types of genera is possible, and names can be protected so that they cannot be replaced by older but later discovered synonyms. Careful consideration and restraint are required at the taxonomic research side, but the clinical user also has to accept the reality that frequently more than one name may persist in the literature for the same fungus for quite some time to come. As long as there is no consensus, some laboratories mention old and new names in their reports. Kidd et al. (12) suggested a 5-y transition period.

Nomenclatural databases, i.e., *Index Fungorum* (www.indexfungorum.org) and *MycoBank* (www.mycobank.org) apply latest names of new fungi and adjustments but refrain from providing recommendations of the usefulness of the changes. Therefore, a list of recommended names (Table 1; www.atlasclinicalfungi.org/nomenclature) has been proposed with the following considerations: (i) the table covers a list of medically important fungi that are considered most commonly encountered in clinical laboratories based on their inclusion in seven textbooks of medical mycology and two reference resources (CAP master list and Doctor Fungus online reference); (ii) the current recommendations on how to report these fungi in the table is based on the rationale illustrated in the present manuscript and consensus agreement among authors after an extensive consultation; (iii) as clinical laboratories are struggling with how to report these fungi to patient-care providers due to nomenclature variation in the literature, the table may serve as a current reference to guide clinical laboratories on how to report these fungi; (iv) the names listed in the table are not fixed names but rather represent nomenclature stability. We recommend that this list, including our author’s consensus reporting recommendations, could be used to guide initial efforts by an upcoming international committee representative of clinical microbiologists, physicians, medical mycologists, and taxonomists. This committee will function as a governing body to curate future fungal name changes for clinical use. We also recommend special focus on reporting some names (e.g., some *Candida* species) of which the present authors were unable to reach consensus using the methods discussed in this document.

**TABLE 1:** Common and medically important fungi^*a*^

Classical name most commonly used in clinical laboratories^*b*^	Alternative name appeared in literature (anamorph, teleomorph, synonym, synanamorph, or obsolete name)	Recommended name to be reported for clinical use^*c*^
*Acremonium egyptiacum*	*Acremonium sclerotigenum*	*Acremonium egyptiacum*
*Acremonium kiliense*	*Cephalosporium kiliense, Sarocladium kiliense*	*Sarocladium kiliense*
*Acremonium recifei*	*Xenoacremonium recifei*	*Acremonium recifei*
*Acremonium strictum*	*Cephalosporium acremonium, Sarocladium strictum*	*Sarocladium strictum*
*Acrophialophora fusispora*	*Paecilomyces fusisporus*	*Acrophialophora fusispora*
*Acrophialophora levis*		*Acrophialophora levis*
*Actinomucor elegans*	*Mucor elegans, Rhizopus elegans*	*Actinomucor elegans*
*Alternaria alternata*		*Alternaria alternata*
*Alternaria infectoria*		*Alternaria infectoria*
*Alternaria tenuissima*	*Helminthosporium tenuissimum*	*Alternaria tenuissima*
*Aphanoascus keratinophilus*	*Chrysosporium keratinophilum*	*Aphanoascus keratinophilus*
*Apophysomyces elegans*		*Apophysomyces elegans*
*Apophysomyces trapeziformis*		*Apophysomyces trapeziformis*
*Apophysomyces variabilis*		*Apophysomyces variabilis*
*Arthrinium arundinis*	*Apiospora arundinis*	*Arthrinium arundinis*
*Arthrographis kalrae*	*Oididendron kalrae*	*Arthrographis kalrae*
*Aspergillus calidoustus*		*Aspergillus calidoustus*
*Aspergillus flavus*	*Aspergillus oryzae*	*Aspergillus flavus*
*Aspergillus fischeri*	*Aspergillus fischerianus, Neosartorya fischeri*	*Aspergillus fischeri* [member of *A. fumigatus* series (species complex)]
*Aspergillus fumigatus*		*Aspergillus fumigatus*
*Aspergillus glaucus*	*Eurotium herbariorum*	*Aspergillus glaucus*
*Aspergillus lentulus*		*Aspergillus lentulus*
*Aspergillus nidulans*	*Emericella nidulans*	*Aspergillus nidulans*
*Aspergillus niger*		*Aspergillus niger*
*Aspergillus sclerotiorum*		*Aspergillus sclerotiorum*
*Aspergillus sydowii*		*Aspergillus sydowii*
*Aspergillus terreus*		*Aspergillus terreus*
*Aspergillus thermomutatus*	*Aspergillus fischeri* var. *thermomutatus, Neosartorya pseudofischeri*	*Aspergillus thermomutatus*
*Aspergillus tubingensis*		*Aspergillus tubingensis* [member of *A. niger* series (species complex)]
*Aspergillus udagawae*	*Neosartorya udagawae*	*Aspergillus udagawae*
*Aspergillus unguis*	*Emericella unguis*	*Aspergillus unguis*
*Aspergillus ustus*		*Aspergillus ustus*
*Aspergillus versicolor*		*Aspergillus versicolor*
*Aureobasidium melanogenum*	*Aureobasidium pullulans* var. *melanogenum*	*Aureobasidium melanogenum*
*Aureobasidium pullulans*		*Aureobasidium pullulans*
*Basidiobolus ranarum*		*Basidiobolus ranarum*
*Beauveria bassiana*		*Beauveria bassiana*
*Bipolaris australiensis*	*Curvularia australiensis*	*Bipolaris australiensis*
*Bipolaris hawaiiensis*	*Cochliobolus hawaiiensis, Curvularia hawaiiensis*	*Bipolaris hawaiiensis*
*Bipolaris spicifera*	*Drechslera spicifera, Cochliobolus spicifer, Curvularia spicifera*	*Bipolaris spicifera*
*Blastomyces dermatitidis*	*Ajellomyces dermatitidis*	*Blastomyces dermatitidis*
*Blastomyces gilchristii*		*Blastomyces gilchristii* (member of *B. dermatitidis* complex)
*Candida albicans*		*Candida albicans*
*Candida auris*		*Candida auris*
*Candida bracarensis*	*Nakaseomyces bracarensis*	*Candida bracarensis* or *Nakaseomyces bracarensis* (member of *C. glabrata* complex) *^d^*
*Candida dubliniensis*		*Candida dubliniensis*
*Candida duobushaemulonis*	*Candida duobushaemuloni, Candida duobushaemuli*	*Candida duobushaemuli* (member of *C. haemuli* complex)
*Candida fabianii*	*Hansenula fabianii, Pichia fabianii, Lindnera fabianii, Cyberlindnera fabianii*	*Candida fabianii* or *Cyberlindnera fabianii^d^*
*Candida famata*	*Debaryomyces hansenii*	*Candida famata* or *Debaryomyces hansenii^d^*
*Candida fermentati*	*Torula fermentati, Pichia caribbica, Meyerozyma caribbica*	*Candida fermentati* (member of *C. guilliermondii* complex)
*Candida glabrata*	*Torulopsis glabrata, Nakaseomyces glabratus*	*Candida glabrata* or *Nakaseomyces glabratus^d^*
*Candida guilliermondii*	*Pichia guilliermondii, Meyerozyma guilliermondii*	*Candida guilliermondii* or *Meyerozyma guilliermondii^d^*
*Candida guilliermondii var. membranifaciens*	*Pichia ohmeri, Yamadazyma ohmeri, Kodamaea ohmeri*	*Kodamaea ohmeri^d^*
*Candida haemulonis*	*Torulopsis haemulonis, Candida haemuloni, Candida haemuli*	*Candida haemuli* (member of *C. haemuli* complex)
*Candida inconspicua*	*Torulopsis inconspicua, Pichia cactophila*	*Candida inconspicua*
*Candida kefyr*	*Candida pseudotropicalis, Kluyveromyces marxianus*	*Candida kefyr* or *Kluyveromyces marxianus^d^*
*Candida krusei*	*Issatchenkia orientalis, Pichia kudriavzevii*	*Candida krusei* or *Pichia kudriavzevii^d^*
*Candida lambica*	*Pichia fermentans*	*Candida lambica* or *Pichia fermentans^d^*
*Candida lipolytica*	*Yarrowia lipolytica*	*Candida lipolytica* or *Yarrowia lipolytica^d^*
*Candida lusitaniae*	*Clavispora lusitaniae*	*Candida lusitaniae* or *Clavispora lusitaniae^d^*
*Candida metapsilosis*		*Candida metapsilosis* (member of *C. parapsilosis* complex)
*Candida nivariensis*	*Nakaseomyces nivariensis*	*Candida nivariensis* or *Nakaseomyces nivariensis* (member of *C. glabrata* complex) *^d^*
*Candida norvegensis*	*Pichia norvegensis*	*Candida norvegensis* or *Pichia norvegensis^d^*
*Candida orthopsilosis*		*Candida orthopsilosis* (member of *C. parapsilosis complex*)
*Candida parapsilosis*		*Candida parapsilosis*
*Candida pelliculosa*	*Hansenula anomala, Pichia anomala, Wickerhamomyces anomalus*	*Candida pelliculosa* or *Hansenula anomala^d^*
*Candida rugosa*	*Diutina rugosa*	*Candida rugosa* or *Diutina rugosa^d^*
*Candida tropicalis*		*Candida tropicalis*
*Candida utilis*	*Torulopsis utilis, Pichia jadinii, Lindnera jadinii, Cyberlindnera jadinii*	*Candida utilis* or *Cyberlindnera jadinii^d^*
*Chaetomium atrobrunneum*	*Amesia atrobrunnea*	*Chaetomium atrobrunneum*
*Chaetomium brasiliense*	*Ovatospora brasiliensis*	*Chaetomium brasiliense*
*Chaetomium globosum*		*Chaetomium globosum*
*Chaetomium perlucidum*	*Parachaetomium perlucidum*	*Chaetomium perlucidum*
*Chaetomium strumarium*	*Achaetomium strumarium*	*Chaetomium strumarium*
*Chrysonilia sitophila*	*Monila sitophila, Neurospora sitophila*	*Neurospora sitophila*
*Chrysosporium queenslandicum*	*Uncinocarpus queenslandicus, Brunneospora queenslandica*	*Brunneospora queenslandica*
*Chrysosporium zonatum*	*Uncinocarpus orissae*	*Chrysosporium zonatum*
*Cladophialophora bantiana*	*Xylohypha bantiana, Cladosporium bantianum, Cladosporium trichoides*	*Cladophialophora bantiana*
*Cladophialophora boppi*	*Taeniolella boppii*	*Cladophialophora boppii*
*Cladophialophora carrionii*	*Cladosporium carrionii*	*Cladophialophora carrionii*
*Cladosporium cladosporioides*		*Cladosporium cladosporioides*
*Cladosporium herbarum*		*Cladosporium herbarum*
*Cladosporium sphaerospermum*		*Cladosporium sphaerospermum*
*Coccidioides immitis*		*Coccidioides immitis*
*Coccidioides posadasii*		*Coccidioides posadasii*
*Cokeromyces recurvatus*		*Cokeromyces recurvatus*
*Collectotrichum coccodes*		*Collectotrichum coccodes*
*Colletotrichum gloeosporioides*		*Colletotrichum gloeosporioides*
*Conidiobolus coronatus*		*Conidiobolus coronatus*
*Cryptococcus adeliensis*	*Naganishia adeliensis*	*Naganishia adeliensis*
*Cryptococcus albidus*	*Cryptococcus albidus* var. *albidus, C. albidus* var. *diffluens, Cryptococcus diffluens, Cryptococcus genitalis, Naganishia albida*	*Naganishia albida*
*Cryptococcus gattii*		*Cryptococcus gattii*
*Cryptococcus laurentii*	*Torula laurentii, Papiliotrema laurentii*	*Papiliotrema laurentii*
*Cryptococcus liquefaciens*	*Torulopsis liquefaciens, Naganishia liquefaciens*	*Naganishia liquefaciens*
*Cryptococcus neoformans*		*Cryptococcus neoformans*
*Cryptococcus uniguttulatus*	*Filobasidium uniguttulatum*	*Filobasidium uniguttulatum*
*Cunninghamella bertholletiae*		*Cunninghamella bertholletiae*
*Curvularia geniculata*	*Cochliobolus geniculatus*	*Curvularia geniculata*
*Curvularia lunata*	*Cochliobolus lunatus*	*Curvularia lunata*
*Curvularia pallescens*	*Cochliobolus pallescens*	*Curvularia pallescens*
*Cylindrocarpon cyanescens*	*Fusarium cyanescens, Neocosmospora cyanescens*	*Cylindrocarpon cyanescens*
*Cylindrocarpon destructans*	*Ilyonectria destructans*	*Cylindrocarpon destructans*
*Drechslera biseptata*	*Pyrenophora biseptata*	*Drechslera biseptata*
*Emmonsia crescens*	*Emmonsia parva* var. *crescens, Ajellomyces crescens, Emergomyces crescens*	*Emmonsia crescens*
*Emmonsia parva*	*Chrysosporium parvum, Blastomyces parvus*	*Blastomyces parvus*
*Emmonsia pasteuriana*	*Emergomyces pasteurianus*	*Emergomyces pasteurianus*
*Engyodontium album*	*Tritirachium album, Beauveria alba, Parengyodontium album*	*Parengyodontium album*
*Epicoccum nigrum*	*Epicoccum purpurascens, Phoma epicoccina*	*Epicoccum nigrum*
*Epidermophyton floccosum*	*Acrothecium floccosum, Trichothecium floccosum*	*Epidermophyton floccosum*
*Exophiala dermatitidis*	*Wangiella dermatitidis*	*Exophiala dermatitidis*
*Exophiala jeanselmei*		*Exophiala jeanselmei*
*Exophiala lecanii-corni*	*Exophiala jeanselmei* var. *lecanii-corni*	*Exophiala lecanii-corni*
*Exophiala oligosperma*		*Exophiala oligosperma*
*Exophiala spinifera*	*Phialophora spinifera, Rhinocladiella spinifera*	*Exophiala spinifera*
*Exserohilum rostratum*	*Helminthosporium rostratum, Exserohilum longirostratum, Exserohilum macginnisii*	*Exserohilum rostratum*
*Fonsecaea monophora*		*Fonsecaea monophora*
*Fonsecaea pedrosoi*	*Fonsecaea compacta*	*Fonsecaea pedrosoi*
*Fusarium chlamydosporum*		*Fusarium chlamydosporum*
*Fusarium dimerum*	*Bisifusarium dimerum*	*Fusarium dimerum*
*Fusarium falciforme*	*Acremonium falciforme, Neocosmospora falciforme*	*Fusarium falciforme* (member of *F. solani* complex)
*Fusarium incarnatum*	*Fusarium semitectum*	*Fusarium incarnatum* (member of *F. incarnatum-equiseti* complex)
*Fusarium keratoplasticum*	*Neocosmospora keratoplastica*	*Fusarium keratoplasticum* (member of *F. solani* complex)
*Fusarium lichenicola*	*Cylindrocarpon lichenicola, Neocosmospora lichenicola*	*Fusarium lichenicola* (member of *F. solani* complex)
*Fusarium neocosmosporiellum*	*Neocosmospora vasinfecta*	*Fusarium neocomosporiellum* (member of *F. solani* complex)
*Fusarium oxysporum*		*Fusarium oxysporum*
*Fusarium petroliphilum*	*Neocosmospora petroliphila*	*Fusarium petroliphilum* (member of *F. solani* complex)
*Fusarium proliferatum*		*Fusarium proliferatum* (member of *F. fujikuroi* complex)
*Fusarium solani*	*Neocosmospora solani*	*Fusarium solani*
*Fusarium verticillioides*	*Fusarium moniliforme, Gibberella moniliformis*	*Fusarium verticillioides* (member of *F. fujikuroi* complex)
*Geomyces destructans*	*Pseudogymnoascus destructans*	*Pseudogymnoascus destructans*
*Geomyces pannorum*	*Chrysosporium pannorum, Pseudogymnoascus pannorum*	*Geomyces pannorum*
*Geotrichum candidum*	*Galactomyces candidus, Dipodascus geotrichum*	*Geotrichum candidum*
*Geotrichum capitatum*	*Dipodascus capitatus, Blastoschizomyces capitatus, Saprochaetea capitata, Magnusiomyces capitatus*	*Magnusiomyces capitatus*
*Geotrichum clavatum*	*Saprochaete clavate, Magnusiomyces clavatus*	*Magnusiomyces clavatus*
*Gymnascella hyalinospora*	*Narasimhella hyalinospora, Gymnoascus hyalinosporus*	*Gymnascella hyalinospora*
*Histoplasma capsulatum*	*Ajellomyces capsulatus*	*Histoplasma capsulatum*
*Hormographiella aspergillata*	*Coprinus cinereus, Coprinopsis cinerea*	*Coprinopsis cinerea*
*Hormographiella verticillata*	*Coprinus domesticus, Coprinellus domesticus*	*Coprinellus domesticus*
*Hortaea werneckii*	*Exophiala werneckii, Phaeoannellomyces werneckii*	*Hortaea werneckii*
*Lasiodiplodia theobromae*	*Botryosphaeria rhodina*	*Lasiodiplodia theobromae*
*Lecythophora hoffmannii*	*Coniochaeta hoffmannii*	*Lecythophora hoffmannii*
*Lecythophora mutabilis*	*Coniochaeta mutabilis*	*Lecythophora mutabilis*
*Lichtheimia corymbifera*	*Absidia corymbifera*	*Lichtheimia corymbifera*
*Lodderomyces elongisporus*	*Saccharomyces elongisporus*	*Lodderomyces elongisporus^d^*
*Lomentospora prolificans*	*Scedosporium prolificans, Scedosporium inflatum*	*Lomentospora prolificans*
*Madurella grisea*	*Trematosphaeria grisea*	*Trematosphaeria grisea*
*Madurella mycetomatis*	*Madurella mycetomi*	*Madurella mycetomatis*
*Malassezia furfur*		*Malassezia furfur*
*Malassezia globosa*		*Malassezia globosa*
*Malassezia pachydermatis*		*Malassezia pachydermatis*
*Malassezia restricta*		*Malassezia restricta*
*Malassezia slooffiae*		*Malassezia slooffiae*
*Malassezia sympodialis*		*Malassezia sympodialis*
*Malbranchea pulchella*		*Malbranchea pulchella*
*Microascus cinereus*	*Scopulariopsis cinereus*	*Microascus cinereus*
*Microascus cirrosus*		*Microascus cirrosus*
*Microsporum audouinii*		*Microsporum audouinii*
*Microsporum canis*	*Microsporum canis* var. *distortum, Arthroderma otae, Microsporum equinum*	*Microsporum canis*
*Microsporum cookei*	*Paraphyton cookei*	*Paraphyton cookei*
*Microsporum ferrugineum*		*Microsporum ferrugineum*
*Microsporum gallinae*	*Lophophyton gallinae*	*Lophophyton gallinae*
*Microsporum gypseum*	*Nannizzia gypsea*	*Nannizzia gypsea*
*Microsporum nanum*	*Arthroderma obtusum, Nannizzia nana*	*Nannizzia nana*
*Microsporum persicolor*	*Arthroderma persicolor, Nannizzia persicolor*	*Nannizzia persicolor*
*Mortierella wolfii*	*Actinomortierella wolfii*	*Mortierella wolfii*
*Mucor circinelloides*		*Mucor circinelloides*
*Mucor indicus*		*Mucor indicus*
*Mucor irregularis*	*Rhizomucor variabilis*	*Mucor irregularis*
*Mucor ramosissimus*		*Mucor ramosissimus* (member of *M. circinelloides* complex)
*Mucor velutinosus*		*Mucor velutinosus* (member of *M. circinelloides* complex)
*Myceliophthora thermophila*	*Chrysosporium thermophilum, Thermothelomyces thermophila*	*Myceliophthora thermophila*
*Nigrospora sphaerica*		*Nigrospora sphaerica*
*Nodulisporium griseobrunneum*	*Hypoxylon griseobrunneum*	*Hypoxylon griseobrunneum*
*Ochroconis gallopava*	*Dactylaria gallopava, Dactylaria constricta* var. *gallopava, Verruconis gallopava*	*Verruconis gallopava*
*Ochroconis mirabilis*	*Ochroconis musae, Scolecobasidium musae, Scolecobasidium mirabilis*	*Scolecobasidium mirabilis*
*Onychocola canadensis*	*Arachnomyces nodosetosus*	*Arachnomyces nodosetosus*
*Paecilomyces formosus*		*Paecilomyces formosus*
*Paecilomyces lilacinus*	*Purpureocillium lilacinum*	*Purpureocillium lilacinum*
*Paecilomyces variotii*	*Paecilomyces spectabilis, Byssochlamys spectabilis*	*Paecilomyces variotii*
*Paracoccidioides brasiliensis*		*Paracoccidioide brasiliensis*
*Paracoccidioides lutzii*		*Paracoccidioides lutzii* (member of *P. brasiliensis* complex)
*Penicillium chrysogenum*		*Penicillium chrysogenum*
*Penicillium citrinum*		*Penicillium citrinum*
*Penicillium marneffei*	*Talaromyces marneffei*	*Talaromyces marneffei*
*Penicillium purpureogenum*	*Talaromyces purpureogenus*	*Talaromyces purpureogenus*
*Phaeoacremonium parasiticum*	*Phialophora parasitica, Togninia parasitica*	*Phaeoacremonium parasiticum*
*Phialemonium curvatum*	*Phialemonium dimorphosporum, Thyridium curvatum, Phialemoniopsis curvata*	*Thyridium curvatum*
*Phialemonium atrogriseum*	*Acremonium atrogriseum*	*Phialemonium atrogriseum*
*Phialemonium obovatum*		*Phialemonium obovatum*
*Phialophora americana*	*Capronia semiimmersa, Cadophora americana*	*Phialophora americana*
*Phialophora europaea*	*Cyphellophora europaea*	*Cyphellophora europaea*
*Phialophora richardsiae*	*Pleurostomophora richardsiae, Pleurostoma richardsiae*	*Pleurostoma richardsiae*
*Phialophora verrucosa*		*Phialophora verrucosa*
*Phoma cruris-hominis*		*Phoma cruris-hominis*
*Phoma herbarum*	*Phoma muscivora*	*Phoma herbarum*
*Piedraia hortae*		*Piedraia hortae*
*Pithomyces chartarum*	*Pseudopithomyces chartarum*	*Pithomyces chartarum*
*Prototheca wickerhamii*		*Prototheca wickerhamii^e^*
*Pseudozyma aphidis*	*Moesziomyces aphidis*	*Moesziomyces aphidis*
*Pyrenochaeta romeroi*	*Medicopsis romeroi*	*Medicopsis romeroi*
*Pythium insidiosum*		*Pythium insidiosum^e^*
*Ramichloridium schulzeri*	*Myrmecridium schulzeri*	*Myrmecridium schulzeri*
*Rasamsonia aegroticola*		*Rasamsonia aegroticola*
*Rasamsonia argillacea*	*Penicillum argillaceum, Geosmithia argillacea*	*Rasamsonia argillacea* (member of *R. argillacea* complex)
*Rhinocladiella aquaspersa*		*Rhinocladiella aquaspersa*
*Rhinocladiella mackenziei*	*Ramichloridium mackenziei*	*Rhinocladiella mackenziei*
*Rhinocladiella similis*		*Rhinocladiella similis*
*Rhizomucor miehei*		*Rhizomucor miehei*
*Rhizomucor pusillus*		*Rhizomucor pusillus*
*Rhizopus arrhizus*	*Rhizopus oryzae*	*Rhizopus arrhizus*
*Rhizopus azygosporus*		*Rhizopus azygosporus*
*Rhizopus microsporus*	*Rhizopus rhizopodiformis*	*Rhizopus microsporus*
*Rhizopus schipperae*		*Rhizopus schipperae*
*Rhizopus stolonifer*		*Rhizopus stolonifer*
*Rhodotorula glutinis*	*Torulopsis glutinis*	*Rhodotorula glutinis*
*Rhodotorula minuta*	*Torula minuta, Cystobasidium minutum*	*Rhodotorula minuta*
*Rhodotorula mucilaginosa*	*Rhodotorula rubra, Torula mucilaginosa*	*Rhodotorula mucilaginosa*
*Saccharomyces cerevisiae*	*Saccharomyces boulardii*	*Saccharomyces cerevisiae*
*Saksenaea vasiformis*		*Saksenaea vasiformis*
*Scedosporium apiospermum*	*Pseudallescheria apiosperma*	*Scedosporium apiospermum* (member of *S. apiospermum* complex)
*Scedosporium aurantiacum*		*Scedosporium aurantiacum*
*Scedosporium boydii*	*Pseudallescheria boydii*	*Scedosporium boydii* (member of *S. apiospermum* complex)
*Schizophyllum commune*		*Schizophyllum commune*
*Schizophyllum radiatum*		*Schizophyllum radiatum*
*Scopulariopsis brevicaulis*	*Microascus brevicaulis*	*Scopulariopsis brevicaulis*
*Scopulariopsis brumptii*	*Microascus paisii*	*Scopulariopsis brumptii*
*Scytalidium dimidiatum*	*Hendersonula toruloidea, Nattrassia mangiferae, Neoscytalidium dimidiatum*	*Neoscytalidium dimidiatum*
*Sporobolomyces salmonicolor*	*Sporidiobolus salmonicolor*	*Sporobolomyces salmonicolor*
*Sporothrix brasiliensis*		*Sporothrix brasiliensis*
*Sporothrix cyanescens*	*Fugomyces cyanescens, Cerinosterus cyanescens, Quambalaria cyanescens*	*Quambalaria cyanescens*
*Sporothrix globosa*		*Sporothrix globosa*
*Sporothrix luriei*	*Sporothrix schenckii* var. *luriei*	*Sporothrix luriei*
*Sporothrix mexicana*		*Sporothrix mexicana*
*Sporothrix schenckii*		*Sporothrix schenckii*
*Sporotrichum pruinosum*	*Chrysosporium pruinosum, Phanerochaete chrysosporium*	*Phanerochaete chrysosporium*
*Syncephalastrum racemosum*		*Syncephalastrum racemosum*
*Trichoderma harzianum*		*Trichoderma harzianum*
*Trichoderma longibrachiatum*		*Trichoderma longibrachiatum*
*Trichophyton benhamiae*	*Arthroderma benhamiae*	*Trichophyton benhamiae*
*Trichophyton concentricum*		*Trichophyton concentricum* (member of *T. benhamiae* complex)
*Trichophyton equinum*		*Trichophyton equinum* (member of *T. tonsurans* complex)
*Trichophyton indotineae*	*Trichophyton mentagrophytes* ITS Type VIII	*Trichophyton indotineae*
*Trichophyton interdigitale*	*Trichophyton krajdenii*	*Trichophyton interdigitale* (member of *T. mentagrophytes* complex)
*Trichophyton mentagrophytes*		*Trichophyton mentagrophytes*
*Trichophyton rubrum*		*Trichophyton rubrum*
*Trichophyton schoenleinii*		*Trichophyton schoenleinii*
*Trichophyton soudanense*		*Trichophyton soudanense* (member of *T. rubrum* complex)
*Trichophyton tonsurans*		*Trichophyton tonsurans*
*Trichophyton verrucosum*		*Trichophyton verrucosum*
*Trichophyton violaceum*	*Trichophyton yaoundei*	*Trichophyton violaceum* (member of *T. rubrum* complex)
*Trichosporon asahii*		*Trichosporon asahii*
*Trichosporon dermatis*	*Cutaneotrichosporon dermatis*	*Trichosporon dermatis* or *Cutaneotrichosporon dermatis^d^*
*Trichosporon inkin*		*Trichosporon inkin*
*Trichosporon loubieri*	*Apiotrichum loubieri*	*Apiotrichum oubieri*
*Trichosporon mucoides*	*Cutaneotrichosporon mucoides*	*Trichosporon mucoides* or *Cutaneotrichosporon mucoides^d^*
*Trichosporon mycotoxinivorans*	*Apiotrichum mycotoxinivorans*	*Apiotrichum mycotoxinivorans*
*Tritirachium oryzae*	*Beauveria oryzae, Tritirachium roseum*	*Tritirachium oryzae*
*Ulocladium botrytis*	*Alternaria botrytis*	*Alternaria botrytis*
*Ulocladium chartarum*	*Alternaria chartarum*	*Alternaria chartarum*
*Veronaea botryosa*		*Veronaea botryosa*
*Verticillium dahliae*		*Verticillium dahliae*

^*a*^ We recommend Table 1 for the following reasons: (i) the table covers a list of medically important fungi that are considered as most commonly encountered in the clinical labs and listed in seven Medical Mycology Textbooks widely used in the clinical laboratories; (ii) the current recommendation on how to report these fungi in the table is based on the rationale illustrated in the manuscript and consensus agreement among authors after an extensive review; (3) as clinical laboratories are struggling with how to report these fungi to clinicians due to nomenclature variation in the literature, the table may serve as a current reference to guide clinical laboratories on how to report these fungi; (4) the names listed in the table are not fixed names but rather representing nomenclature stability; they will be reviewed and updated periodically by an international committee representative of clinical microbiologists, physicians, medical mycologists, and taxonomists. Note: a complete overview of pathogenic and opportunistic species with descriptions and references can be found in Atlas of Clinical Fungi, 4th ed. 2020.

^*b*^ Based on a list of textbooks and reference materials used in clinical labs: (i) Larone’s Medically Important fungi 6th Ed by Thomas J Walsh, Randall T. Hayden, Davise H. Larone; (ii) Manual of Clinical Microbiology 12th Ed; (iii) CAP (College of American Pathologists) Master list of Fungi; (iv) CAP Color Atlas of Mycology by Gordon L. Love, Julie A. Ribes; (v) Guide to Clinically Significant Fungi by Deanna A. Sutton, Annette W. Fothergill, Michael G. Rinaldi; (vi) Doctor Fungus online reference (https://drfungus.org/knowledge-base-category/fungi-descriptions/); (vii) Identifying fungi: a clinical laboratory handbook 2nd Ed by Guy St-Germain, Richard Summerbell; (viii) Identification of pathogenic fungi 2nd Ed by Colin K. Campbell, Elizabeth M. Johnson, David W. Warnock; (ix) Descriptions of medical fungi 4th Ed by Sarah Kidd, Catriona Halliday, David Ellis. Furthermore, only the ones that have been reported causing human infection are included.

^*c*^ The recommended names to be reported for clinical use are based on current treatment guidelines and the Atlas of Clinical Fungi (https://www.clinicalfungi.org). The fungi listed in the table represent a list of the common and medically important ones from the database of the ATLAS (>6,000 clinical fungi). Species level identification on some of them may not be readily achievable based on morphological or phenotypic features and thus will have to rely on additional tools, e.g., DNA sequencing identification or MALDI-TOF MS to obtain reliable species identification.

^*d*^ These names will be further reviewed by an international committee representative of clinical microbiologists, physicians, medical mycologists, and taxonomists.

^*e*^ Not a fungus.

Incorporating name changes that are validated and stable is essential for clinical laboratories and patient care. Other well-established groups which review relevant scientific advances for application in clinical laboratories include the Clinical and Laboratory Standards Institute (CLSI) and the European Committee on Antimicrobial Susceptibility Testing (EUCAST). These organizations provide additional levels of standardization and validation of diagnostic changes before introduction into clinical laboratories. Clinical practice will benefit from clear guidance from the international community on the adaption to nomenclatural changes of medically important fungi. Future association with the database of SNOMED CT and LONIC codes might be considered, thereby facilitating international digital data exchange and standardization of patient reports.

We recognize it is important to disseminate nomenclature changes to clinical community timely and accurately. Education required not only for the clinician but also for the taxonomist that has an insufficient eye for the user. Published lists are an alternative but are forgotten within a few years. We, therefore, think that the most parsimonious and sustainable solution is a readily accessible database, which is easy to find and easy to handle. Only a single website should be remembered. The contents of the database will be supervised by a committee of ISHAM (International Society for Human and Animal Mycology), composed of researchers, as well as of users, and decisions are made in consultancy with taxonomic specialists. Furthermore, the committee will partner with the societies that support this document and use their proper media channels to deliver any recommended nomenclature changes to the clinicians and clinical laboratories widely without any delay.

## References

[1] Redhead SA . 2010. Report on the special committee on the nomenclature of fungi with a pleomorphic life cycle. TAXON 59:1863–1866. doi:10.1002/tax.596017

[2] Hawksworth DL , Crous PW , Redhead SA , Reynolds DR , Samson RA , Seifert KA , Taylor JW , Wingfield MJ , Abaci O , Aime C , et al. . 2011. The Amsterdam declaration on fungal nomenclature. IMA Fungus 2:105–112. doi:10.5598/imafungus.2011.02.01.14 22679594 PMC3317370

[3] Flann C , Turland NJ , Monro AM . 2014. Report on botanical nomenclature—Melbourne 2011. XVIII international botanical congress, Melbourne: nomenclature section, 18–22 July 2011. PhytoKeys:1–289. doi:10.3897/phytokeys.41.8398 PMC4154305 25197229

[4] McNeill J , Barrie F , Buck W , Demoulin V , We G , Hawksworth D , Herendeen P , Knapp S , Marhold K , Prado J . 2012. International code of nomenclature for algae, fungi and plants (Melbourne code). Vol. 154. Koeltz scientific books, Königstein.

[5] Guarro J , Gené J , Ahmed SA , Al-Hatmi AMS , Figueras MJ , Vitale RG . 2020. Atlas of clinical fungi. 4th ed, p 1599. Foundation Atlas of Clinical Fungi, Hilversum.

[6] Chen M , Zeng J , De Hoog GS , Stielow B , Gerrits Van Den Ende AHG , Liao W , Lackner M . 2016. The ‘species complex’ issue in clinically relevant fungi: a case study in Scedosporium apiospermum. Fungal Biol 120:137–146. doi:10.1016/j.funbio.2015.09.003 26781369

[7] de Hoog GS , Chaturvedi V , Denning DW , Dyer PS , Frisvad JC , Geiser D , Gräser Y , Guarro J , Haase G , Kwon-Chung K-J , Meis JF , Meyer W , Pitt JI , Samson RA , Taylor JW , Tintelnot K , Vitale RG , Walsh TJ , Lackner M , ISHAM Working Group on Nomenclature of Medical Fungi . 2015. Name changes in medically important fungi and their implications for clinical practice. J Clin Microbiol 53:1056–1062. doi:10.1128/JCM.02016-14 25297326 PMC4365198

[8] de Hoog GS , Haase G , Chaturvedi V , Walsh TJ , Meyer W , Lackner M . 2013. Taxonomy of medically important fungi in the molecular era. Lancet Infect Dis 13:385–386. doi:10.1016/S1473-3099(13)70058-6 23618329

[9] Kwon-Chung KJ , Bennett JE , Wickes BL , Meyer W , Cuomo CA , Wollenburg KR , Bicanic TA , Castañeda E , Chang YC , Chen J , et al. . 2017. The case for adopting the “species complex” nomenclature for the etiologic agents of cryptococcosis. mSphere 2:e00357-16. doi:10.1128/mSphere.00357-16 PMC5227069 28101535

[10] Yurkov A , Alves A , Bai F-Y , Boundy-Mills K , Buzzini P , Čadež N , Cardinali G , Casaregola S , Chaturvedi V , Collin V , et al. . 2021. Nomenclatural issues concerning cultured yeasts and other fungi: why it is important to avoid unneeded name changes. IMA Fungus 12:18. doi:10.1186/s43008-021-00067-x 34256869 PMC8278710

[11] Borman AM , Johnson EM . 2021. Name changes for fungi of medical importance, 2018 to 2019. J Clin Microbiol 59:e01811-20. doi:10.1128/JCM.01811-20 33028600 PMC8111128

[12] Kidd SE , Abdolrasouli A , Hagen F . 2023. Fungal nomenclature: managing change is the name of the game. Oxford University Press, USA. 10.1093/ofid/ofac559 PMC9825814 36632423

[13] Kidd SE , Halliday CL , McMullan B , Chen S-A , Elvy J . 2021. New names for fungi of medical importance: can we have our cake and eat it too. J Clin Microbiol 59:e02730-20. doi:10.1128/JCM.02730-20 33268539 PMC8106714

[14] Schoch CL , Sung G-H , López-Giráldez F , Townsend JP , Miadlikowska J , Hofstetter V , Robbertse B , Matheny PB , Kauff F , Wang Z , Gueidan C , et al. . 2009. The ascomycota tree of life: a phylum-wide phylogeny clarifies the origin and evolution of fundamental reproductive and ecological traits. Syst Biol 58:224–239. doi:10.1093/sysbio/syp020 20525580

[15] Samson RA , Yilmaz N , Houbraken J , Spierenburg H , Seifert KA , Peterson SW , Varga J , Frisvad JC . 2011. Phylogeny and nomenclature of the genus Talaromyces and taxa accommodated in Penicillium subgenus Biverticillium. Stud Mycol 70:159–183. doi:10.3114/sim.2011.70.04 22308048 PMC3233910

[16] de Hoog GS , Dukik K , Monod M , Packeu A , Stubbe D , Hendrickx M , Kupsch C , Stielow JB , Freeke J , Göker M , Rezaei-Matehkolaei A , Mirhendi H , Gräser Y . 2017. Toward a novel multilocus phylogenetic taxonomy for the dermatophytes. Mycopathologia 182:5–31. doi:10.1007/s11046-016-0073-9 27783317 PMC5283515

[17] Weijman AC . 1979. Carbohydrate composition and taxonomy of Geotrichum, Trichosporon and allied genera. Antonie Van Leeuwenhoek 45:119–127. doi:10.1007/BF00400785 575971

[18] de Beer ZW , Begerow D , Bauer R , Pegg GS , Crous PW , Wingfield MJ . 2006. Phylogeny of the Quambalariaceae fam. nov., including important eucalyptus pathogens in South Africa and Australia. Stud Mycol 55:289–298. doi:10.3114/sim.55.1.289 18490987 PMC2104727

[19] Sugita R , Tanaka K . 2022. Thyridium revised: synonymisation of Phialemoniopsis under Thyridium and establishment of a new order, thyridiales. MycoKeys 86:147–176. doi:10.3897/mycokeys.86.78989 35145340 PMC8825628

[20] Connally A , Smith D , Marek S , Wu Y , Walker N . 2022. Phylogenetic evaluation of Bipolaris and Curvularia species collected from turfgrasses. Int Turfgrass Soc Res J 14:916–930. doi:10.1002/its2.16

[21] Manamgoda DS , Cai L , McKenzie EHC , Crous PW , Madrid H , Chukeatirote E , Shivas RG , Tan YP , Hyde KD . 2012. A phylogenetic and taxonomic re-evaluation of the Bipolaris-Cochliobolus-Curvularia complex. Fungal Div 56:131–144. doi:10.1007/s13225-012-0189-2

[22] Wang XW , Houbraken J , Groenewald JZ , Meijer M , Andersen B , Nielsen KF , Crous PW , Samson RA . 2016. Diversity and taxonomy of Chaetomium and chaetomium-like fungi from indoor environments. Stud Mycol 84:145–224. doi:10.1016/j.simyco.2016.11.005 28082757 PMC5226397

[23] Zachos FE . 2016. Species concepts in biology. Vol. 801. Springer. doi:10.1007/978-3-319-44966-1

[24] Anzawa K , Kawasaki M , Mochizuki T , Ishizaki H . 2010. Successful mating of trichophyton rubrum with Arthroderma simii. Med Mycol 48:629–634. doi:10.3109/13693780903437884 20392147

[25] Hubka V , Barrs V , Dudová Z , Sklenář F , Kubátová A , Matsuzawa T , Yaguchi T , Horie Y , Nováková A , Frisvad JC , Talbot JJ , Kolařík M . 2018. Unravelling species boundaries in the Aspergillus viridinutans complex (section Fumigati): opportunistic human and animal pathogens capable of interspecific hybridization. Persoonia 41:142–174. doi:10.3767/persoonia.2018.41.08 30728603 PMC6344812

[26] Hartmann FE , Duhamel M , Carpentier F , Hood ME , Foulongne-Oriol M , Silar P , Malagnac F , Grognet P , Giraud T . 2021. Recombination suppression and evolutionary strata around mating‐type loci in fungi: documenting patterns and understanding evolutionary and mechanistic causes. New Phytol 229:2470–2491. doi:10.1111/nph.17039 33113229 PMC7898863

[27] Yadav V , Sun S , Heitman J . 2021. Uniparental nuclear inheritance following bisexual mating in fungi. Elife 10:e66234. doi:10.7554/eLife.66234 34338631 PMC8412948

[28] Zhao L , de Hoog S , Hagen F , Kang Y , Al-Hatmi AMS . 2019. Species borderlines in Fusarium exemplified by F. circinatum/F. subglutinans. Fungal Genet Biol 132:103262. doi:10.1016/j.fgb.2019.103262 31415905

[29] Samarasinghe H , Xu J . 2018. Hybrids and hybridization in the Cryptococcus neoformans and Cryptococcus gattii species complexes. Infect Genet Evol 66:245–255. doi:10.1016/j.meegid.2018.10.011 30342094

[30] Samarasinghe H , You M , Jenkinson TS , Xu J , James TY . 2020. Hybridization facilitates adaptive evolution in two major fungal pathogens. Genes 11:101. doi:10.3390/genes11010101 31963231 PMC7017293

[31] Wilson AM , Wilken PM , van der Nest MA , Steenkamp ET , Wingfield MJ , Wingfield BD . 2015. Homothallism: an umbrella term for describing diverse sexual behaviours. IMA Fungus 6:207–214. doi:10.5598/imafungus.2015.06.01.13 26203424 PMC4500084

[32] Stielow JB , Lévesque CA , Seifert KA , Meyer W , Iriny L , Smits D , Renfurm R , Verkley GJM , Groenewald M , Chaduli D , et al. . 2015. One fungus, which genes? Development and assessment of universal primers for potential secondary fungal DNA barcodes. Persoonia 35:242–263. doi:10.3767/003158515X689135 26823635 PMC4713107

[33] Sandoval-Denis M , Sutton DA , Martin-Vicente A , Cano-Lira JF , Wiederhold N , Guarro J , Gené J . 2015. Cladosporium species recovered from clinical samples in the United States. J Clin Microbiol 53:2990–3000. doi:10.1128/JCM.01482-15 26179305 PMC4540897

[34] Wang MM , Crous PW , Sandoval-Denis M , Han SL , Liu F , Liang JM , Duan WJ , Cai L . 2022. Fusarium and allied genera from China: species diversity and distribution. Persoonia 48:1–53. doi:10.3767/persoonia.2022.48.01 PMC10792286 38234691

[35] Hudson O , Waliullah S , Fulton JC , Ji P , Dufault NS , Keinath A , Ali ME . 2021. Marker development for differentiation of Fusarium oxysporum f. sp. niveum race 3 from races 1 and 2. Int J Molec Sci 22:822. doi:10.3390/ijms22020822 33467563 PMC7830397

[36] Vlaardingerbroek I , Beerens B , Schmidt SM , Cornelissen BJC , Rep M . 2016. Dispensable chromosomes in Fusarium oxysporum f. sp. lycopersici. Mol Plant Pathol 17:1455–1466. doi:10.1111/mpp.12440 27271322 PMC6638487

[37] Esposto MC , Prigitano A , Tortorano AM . 2016. Fusarium musae as cause of superficial and deep-seated human infections. J Mycol Med 26:403–405. doi:10.1016/j.mycmed.2016.02.021 27091579

[38] Thitla T , Kumla J , Khuna S , Lumyong S , Suwannarach N . 2022. Species diversity, distribution, and phylogeny of Exophiala with the addition of four new species from Thailand. J Fungi 8:766. doi:10.3390/jof8080766 PMC9331753 35893134

[39] Verbeke V , Bourgeois T , Lodewyck T , Van Praet J , Lagrou K , Reynders M , Nulens E . 2020. Successful outcome of disseminated Fusarium musae fungemia with skin localization treated with liposomal amphotericin B and voriconazole in a patient with acute myeloid leukemia. Mycopathologia 185:1085–1089. doi:10.1007/s11046-020-00499-w 33119817

[40] Gräser Y , De Hoog S , Summerbell RC . 2006. Dermatophytes: recognizing species of clonal fungi. Med Mycol 44:199–209. doi:10.1080/13693780600606810 16702098

[41] Taylor JW , Jacobson DJ , Kroken S , Kasuga T , Geiser DM , Hibbett DS , Fisher MC . 2000. Phylogenetic species recognition and species concepts in fungi. Fungal Genet Biol 31:21–32. doi:10.1006/fgbi.2000.1228 11118132

[42] Kandemir H , Dukik K , Hagen F , Ilkit M , de Hoog G . 2019. A Polyphasic approach for the differentiation Trichophyton tonsurans and T. Equinum. Mycopathologia 185:113–122. 31278475 10.1007/s11046-019-00344-9

[43] Kosanke S , Hamann L , Kupsch C , Moreno Garcia S , Chopra A , Gräser Y . 2018. Unequal distribution of the mating type (MAT) locus idiomorphs in dermatophyte species. Fungal Genet Biol 118:45–53. doi:10.1016/j.fgb.2018.07.003 30016701

[44] Taghipour S , Pchelin IM , Zarei Mahmoudabadi A , Ansari S , Katiraee F , Rafiei A , Shokohi T , Abastabar M , Taraskina AE , Kermani F , Diba K , Nouripour-Sisakht S , Najafzadeh MJ , Pakshir K , Zomorodian K , Ahmadikia K , Rezaei-Matehkolaei A . 2019. Trichophyton mentagrophytes and T interdigitale genotypes are associated with particular geographic areas and clinical manifestations. Mycoses 62:1084–1091. doi:10.1111/myc.12993 31444823

[45] Kuramae EE , Robert V , Echavarri-Erasun C , Boekhout T . 2007. Cophenetic correlation analysis as a strategy to select phylogenetically informative proteins: an example from the fungal kingdom. BMC Evol Biol 7:134. doi:10.1186/1471-2148-7-134 17688684 PMC2045111

[46] van Dam P , de Sain M , Ter Horst A , van der Gragt M , Rep M . 2018. Use of comparative genomics-based markers for discrimination of host specificity in Fusarium oxysporum. Appl Environ Microbiol 84:e01868-17. doi:10.1128/AEM.01868-17 29030446 PMC5734036

[47] Vilela R , de Hoog S , Bensch K , Bagagli E , Mendoza L . 2023. A taxonomic review of the genus Paracoccidioides, with focus on the uncultivable species. PLOS Negl Trop Dis 17:e0011220. doi:10.1371/journal.pntd.0011220 37104274 PMC10138850

[48] Tang C , Ahmed SA , Deng S , Zhang L , Zoll J , Al-Hatmi AMS , Meis JF , Thakur R , Kang Y , de Hoog GS . 2022. Detection of emerging genotypes in Trichophyton mentagrophytes species complex: a proposal for handling biodiversity in dermatophytes. Front Microbiol 13:960190. doi:10.3389/fmicb.2022.960190 36081804 PMC9445586

[49] James TY , Stajich JE , Hittinger CT , Rokas A . 2020. Toward a fully resolved fungal tree of life. Annu Rev Microbiol 74:291–313. doi:10.1146/annurev-micro-022020-051835 32660385

[50] Sandoval-Denis M , Gené J , Sutton DA , Cano-Lira JF , de Hoog GS , Decock CA , Wiederhold NP , Guarro J . 2016. Redefining Microascus, Scopulariopsis and allied genera. Persoonia 36:1–36. doi:10.3767/003158516X688027 27616786 PMC4988368

[51] Kocsubé S , Perrone G , Magistà D , Houbraken J , Varga J , Szigeti G , Hubka V , Hong S-B , Frisvad JC , Samson RA . 2016. Aspergillus is monophyletic: evidence from multiple gene phylogenies and extrolites profiles. Stud Mycol 85:199–213. doi:10.1016/j.simyco.2016.11.006 28082760 PMC5220211

[52] Houbraken J , Kocsubé S , Visagie CM , Yilmaz N , Wang X-C , Meijer M , Kraak B , Hubka V , Bensch K , Samson RA , Frisvad JC . 2020. Classification of Aspergillus, Penicillium, Talaromyces and related genera (Eurotiales): an overview of families, genera, subgenera, sections, series and species. Stud Mycol 95:5–169. doi:10.1016/j.simyco.2020.05.002 32855739 PMC7426331

[53] Quan Y , Muggia L , Moreno LF , Wang M , Al-Hatmi AMS , da Silva Menezes N , Shi D , Deng S , Ahmed S , Hyde KD , Vicente VA , Kang Y , Stielow JB , de Hoog S . 2020. A re-evaluation of the Chaetothyriales using criteria of comparative biology. Fungal Div 103:47–85. doi:10.1007/s13225-020-00452-8

[54] Lücking R , Aime MC , Robbertse B , Miller AN , Ariyawansa HA , Aoki T , Cardinali G , Crous PW , Druzhinina IS , Geiser DM , et al. . 2020. Unambiguous identification of fungi: where do we stand and how accurate and precise is fungal DNA barcoding? IMA Fungus 11:14. doi:10.1186/s43008-020-00033-z 32714773 PMC7353689

[55] Lücking R , Aime MC , Robbertse B , Miller AN , Aoki T , Ariyawansa HA , Cardinali G , Crous PW , Druzhinina IS , Geiser DM , et al. . 2021. Fungal taxonomy and sequence-based nomenclature. Nat Microbiol 6:971. doi:10.1038/s41564-021-00921-z 34045712

[56] Redhead SA . 2001. Bully for Coprinus – a story of manure, minutiae and molecules. Field Mycol 2:118–126. doi:10.1016/S1468-1641(10)60532-4

[57] Bian C , Kusuya Y , Sklenář F , D’hooge E , Yaguchi T , Ban S , Visagie C , Houbraken J , Takahashi H , Hubka V . 2022. Reducing the number of accepted species in Aspergillus series Nigri. Stud Mycol 102:95–132. doi:10.3114/sim.2022.102.03 36760462 PMC9903907

[58] Sklenář F , Glässnerová K , Jurjević Ž , Houbraken J , Samson RA , Visagie CM , Yilmaz N , Gené J , Cano J , Chen AJ , Nováková A , Yaguchi T , Kolařík M , Hubka V . 2022. Taxonomy of Aspergillus series Versicolores: species reduction and lessons learned about intraspecific variability. Stud Mycol 102:53–93. doi:10.3114/sim.2022.102.02 36760461 PMC9903908

[59] Hagen F , Lumbsch HT , Arsic Arsenijevic V , Badali H , Bertout S , Billmyre RB , Bragulat MR , Cabañes FJ , Carbia M , Chakrabarti A , et al. . 2017. Importance of resolving fungal nomenclature: the case of multiple pathogenic species in the Cryptococcus genus. mSphere 2:e00238-17. doi:10.1128/mSphere.00238-17 28875175 PMC5577652

[60] Crous PW , Lombard L , Sandoval-Denis M , Seifert KA , Schroers H-J , Chaverri P , Gené J , Guarro J , Hirooka Y , Bensch K , Kema GHJ , et al. . 2021. Fusarium: more than a node or a foot-shaped basal cell. Stud Mycol 98:100116. doi:10.1016/j.simyco.2021.100116 34466168 PMC8379525

[61] Geiser DM , Al-Hatmi AMS , Aoki T , Arie T , Balmas V , Barnes I , Bergstrom GC , Bhattacharyya MK , Blomquist CL , Bowden RL , et al. . 2021. Phylogenomic analysis of a 55.1-kb 19-gene dataset resolves a monophyletic Fusarium that includes the Fusarium solani species complex. Phytopathology 111:1064–1079. doi:10.1094/PHYTO-08-20-0330-LE 33200960

[62] Geiser DM , Aoki T , Bacon CW , Baker SE , Bhattacharyya MK , Brandt ME , Brown DW , Burgess LW , Chulze S , Coleman JJ , et al. . 2013. One fungus, one name: defining the genus Fusarium in a scientifically robust way that preserves longstanding use. Phytopathology 103:400–408. doi:10.1094/PHYTO-07-12-0150-LE 23379853

[63] O’Donnell K , Al-Hatmi AMS , Aoki T , Brankovics B , Cano-Lira JF , Coleman JJ , de Hoog GS , Di Pietro A , Frandsen RJN , Geiser DM , et al. . 2020. No to Neocosmospora: phylogenomic and practical reasons for continued inclusion of the Fusarium solani species complex in the genus Fusarium. mSphere 5:e00810-20. doi:10.1128/mSphere.00810-20 32938701 PMC7494836

[64] May TW , Redhead SA , Bensch K , Hawksworth DL , Lendemer J , Lombard L , Turland NJ . 2019. Chapter F of the International Code of Nomenclature for algae, fungi, and plants as approved by the 11th International mycological Congress, San Juan, Puerto Rico, July 2018. IMA Fungus 10:21. doi:10.1186/s43008-019-0019-1 32647625 PMC7325661

[65] Turland N , Wiersema J , Barrie F , Greuter W , Hawksworth D , Herendeen P , Knapp S , Kusber W-H , Li D-Z , Marhold K , May T , McNeill J , Monro A , Prado J , Price M , Smith G . 2018. Adopted by the Nineteenth International Botanical Congress. International code of nomenclature for algae, fungi, and plants (shenzhen code). Koeltz Botanical Books, Shenzhen, China. doi:10.12705/Code.2018

